# Sensitivity of Transmission Raman Spectroscopy Signals to Temperature of Biological Tissues

**DOI:** 10.1038/s41598-018-25465-x

**Published:** 2018-05-30

**Authors:** Adrian Ghita, Pavel Matousek, Nick Stone

**Affiliations:** 1 School of Physics and Astronomy, University of Exeter, Streatham Campus, EX4 4QL Exeter, UK; 2 Central Laser Facility, Research Complex at Harwell, STFC Rutherford Appleton Laboratory, Harwell Oxford, OX11 0QX UK

## Abstract

Optical properties of biological tissues can be influenced by their temperature, thus affecting light transport inside the sample. This could potentially be exploited to deliver more photons inside large biological samples, when compared with experiments at room temperature, overcoming some of difficulties due to highly scattering nature of the tissue. Here we report a change in light transmitted inside biological tissue with temperature elevation from 20 to 40 °C, indicating a considerable enhancement of photons collected by the detector in transmission geometry. The measurement of Raman signals in porcine tissue samples, as large as 40 mm in thickness, indicates a considerable increase in signal ranging from 1.3 to 2 fold, subject to biological variability. The enhancements observed are ascribed to phase transitions of lipids in biological samples. This indicates that: 1) experiments performed on tissue at room temperature can lead to an underestimation of signals that would be obtained at depth in the body *in vivo* and 2) that experiments at room temperature could be modified to increase detection limits by elevating the temperature of the material of interest.

## Introduction

Clinical imaging methods used for diagnosis and monitoring of disease are usually reliant on X-rays, ultrasound or magnetic resonance. Of these only magnetic resonance imaging (MRI) can provide molecular information on the tissue/lesion of interest but these devices are highly costly and as such with limited accessibility. Furthermore, prolonged exposure to X-rays may represent a health risk to the patient^1^, due to their ionizing nature. Therefore, there is a considerable gap in the ability of *in vivo* medical diagnostics to provide a widely applicable, detailed chemical analysis of suspicious lesions. In this area, Raman spectroscopy holds considerable prospects for *in-vivo* biological applications as it conveys rich chemical information and water contained in tissue has only a very small influence on signals within the so-called fingerprint region. To date, the vast majority of *in-vivo* applications have concentrated on development of Raman endoscope/probe platforms for various types of diagnostics^2–7^, e.g. for cancer diagnosis intraoperatively to identify tumour margins during surgical excision^8,9^.

There is a strong incentive to use Raman spectroscopy for *in vivo* applications for initial detection and diagnosis as well as monitoring during periodic check-up. It could for example be exploited as a contrast mechanism for patient screening when a particular chemical marker is present and disease occurs. Breast cancer screening, designed for the early detection of signs of calcifications during mammography, can, for example, benefit from such an approach. Breast micro-calcifications are one of the markers used by radiologists as their presence can be sign of early cancers^10^. Two principal types of breast calcification have been identified: hydroxyapatite (HAP or type II) and calcium oxalate (CO or type I)^11^. The calcium oxalate micro-calcifications (type I) have been strongly correlated with the presence of benign features while calcium hydroxyapatite (type II) is found in locations where breast cancers are present as well as benign lesions – however the type of lesions can be further differentiated according to their carbonate content^12^. Currently, breast cancer diagnosis requires that a female patient with suspicious features detected in X-ray images is referred for further tests including excisional biopsy followed by histopathological examination. This financially costly procedure also puts an enormous psychological stress on the patient. However the majority of these referrals are found to be normal^13^. Alternatively, one could contemplate using laser Raman spectroscopy noninvasively to assess the chemistry of calcifications via a transmission setup configuration to complement the X-ray mammography^14^.

The use of light based technologies able to probe molecular constituents using vibrational spectroscopy, such as Raman spectroscopy^15,16^ could address various needs for *in-vivo* diagnostics. However, to achieve these goals, light needs to be delivered effectively into the tissues to depths of many tens of mm (or cm) and Raman molecular vibrational signals detected on the other side. The highly scattering nature of biological tissues makes light propagate in a random walk style, rather than directly (ballistic regime). This leads to a rapid loss of light from the optical axis, meaning only a fraction of the incident light is recovered typically from the other side of tissue. This approach is challenging due to difficulty of light delivery inside turbid media such as biological tissues; as well as the relative weakness of the Raman signal, combined with the high penetration depths required for breast screening (~40–50 mm).

Light propagation in tissue is principally governed by absorption and scattering being described by the coefficients (μ_a_) and (μ_s_’), respectively^17^. If one minimises the effect of absorption inside tissue (e.g. by the appropriate choice of laser excitation wavelength) the scattering process can become the dominant factor contributing to light loss.

In previous work, we published the results of absorption measurements obtained using a modified advanced Transmission Raman setup optimized for detection of HAP inside 40 mm breast phantom tissue (pork). As a results of major instrumental improvements to yield enhanced light collection rates (x110 enhanced Raman photon acquisition rates^14^) the desired sensitivity to detect small amounts of HAP and CO was achieved although this was still challenging within >40–50 mm thick tissues. Crucially, all the spectra reported in our previous paper were measured on phantoms at room temperature (~22 °C); this is significantly lower than the average biological temperature of human breast *in vivo* (~35 °C^18^). Here we explore the dependence of TRS signals on temperature in this important temperature range.

The dynamics of optical properties of tissue associated with temperature represents a topic also of relevance to many biomedical applications such as low level laser therapy^19^. Applications which require light delivery deep inside the tissue for diagnostic purposes can also be affected. A few available papers addressing the temperature influence on the reduced scattering coefficient in biological samples point to a significant variation with temperature^20–23^ observed, in a variety of biological tissue types, that can be correlated with structural adjustments in tissue heterogeneity. At temperatures where protein denaturation occurs a sharp change in scattering coefficient has been reported^24^. It should be noted that all our experiments were performed below 40 °C to avoid any irreversible biochemical processes in tissue.

This paper explores the changes in relative transparency of biological tissues, from the standpoint of transmission Raman spectroscopy (TRS), with temperature. Specifically, when going from room temperature to natural body temperature. To date, the main body of research carried out on excised biological tissues has focused predominantly on studies at room, or lower temperatures. Although some studies did report significant dependence of phantom tissue optical properties on temperature, indicating significant changes in the μ_s_^19,25–28^ value with elevated temperatures and approximate invariance of μ_a_ to temperature the influence of these effects on TRS signals, which involves photon transport of both laser photons as well as inelastically scattered photons, has not been reported to date. It is therefore important to assess the role of temperature in the context of such studies. Here we present preliminary results obtained using a Transmission Raman platform on samples placed in a thermal bath. The Raman spectra were recorded at several temperatures and the results were analysed to assess the influence of temperature on the retrieved TRS signal. The phenomena reported here is not limited to TRS of biological samples. In fact it may play a role also when performing conventional backscattering Raman spectroscopy or imaging of biological tissues.

## Materials and Methods

### Sample preparation

The breast tissue phantoms used in this study were made of porcine shoulder tissues. These contained skin, fat and muscle replicating crudely much of the gross chemical composition of human breast tissue. The tissue samples were purchased fresh from a local supermarket and sliced to a thickness of around 40 mm (illumination path-length) × 35 mm × 50 mm (precision of ±1.5 mm) to fit inside a quartz container. The samples were wrapped in a fine polyethylene cling film to avoid water evaporation during experiments.

Two different amounts of calcium hydroxyapatite (HAP) powder (Sigma Aldrich, St Louise, USA), representing type II breast calcifications^29^, 80 mg and 120 mg were smeared on tissue in the middle of sample over a disk shape area (of approximately 4 mm diameter) oriented perpendicularly to the optical axis.

**Figure 1:**
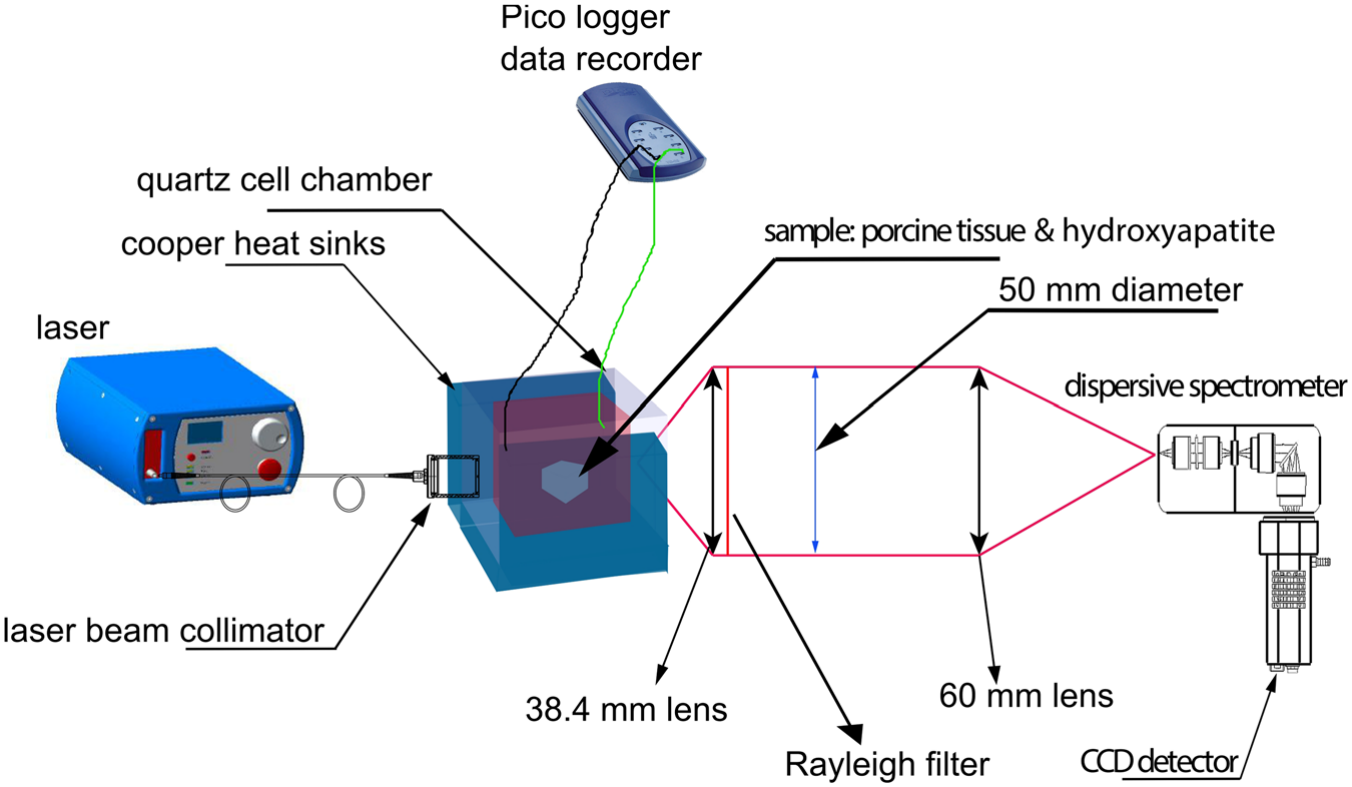
Schematics of the Transmission Raman setup with heated sample holder.

A thermal water bath was used to control sample temperature. The water was piped to a copper based heat exchange element placed underneath, on the left and on right of the sample (see Fig. 1) permitting a uniform and steady heating of samples to desired temperatures.

### Transmission Raman Instrument

The schematic of the TRS setup is presented in Fig. 1 with a detailed description given in our earlier paper^30^. The laser used in these experiments was a solid state laser operating at 808 nm (Innovative Photonic Solutions, Monmouth Junction, NJ, US) and the light was delivered to the sample using an optical fibre (550 µm diameter, 0.22 N.A., Thorlabs, USA). Prior to spectral filtering the laser output was expanded and collimated to 6–8 mm diameter using a biconvex lens with 25 mm focal length and 25 mm diameter. The Raman signal was collected on the other side of the sample with a 50 mm diameter biconvex collection lens of 38 mm focal length (N.A. = 0.6) which directed the light towards relaying lens of the same diameter and a focal length of 60 mm to deliver the light inside spectrometer. A couple of long pass edge filters at 830 nm (LP02-830RU-25, Semrock, Rochester, US) were deployed to filter out (O.D. 7) the elastically scattered light, first being placed between collection and focusing lens and the second located inside the spectrometer. The spectrometer employed in the setup, Holospec 1.8i (Kaiser Optical Systems, Ann Arbor, Michigan, USA) was equipped with a high dispersion grating covering a spectral range of 608–1243 cm^−1^. A deep depletion CCD detector, Andor iDus 420 (Andor, Belfast, UK) was coupled with the spectrometer to collect the Raman signals. The slit used inside the spectrometer was 1 mm wide, which, combined with a high resolution diffraction grating, provided a spectral resolution of ∼15 cm^−1^. Some additional measurements of tissue chemical evolution with temperature were performed with a low dispersion grating and a 50 microns slit (spectral resolution around 12 cm^−1^) to enable the recording of a more precise descriptive picture of tissue dynamics. This is noted in the text.

### Measurements of the absorption profile of tissue

For the measurements of the tissue attenuation profile, the recorded exposure times were 0.1 s with 300 accumulations. A HL-2000 (Ocean Optic, Dunedin, US) white light source was delivered via 400 µm diameter multimode fibre to a 35 mm focal length/diameter 1 inch lens which expanded the beam to 12 mm diameter while a Holospec 1.8i (Kaiser optical, UK) spectrometer was placed on the detection side. For these experiments we used a different custom transmission diffraction grating covering a wider spectral range from 600 nm to 1200 nm in combination with a 50 microns slit.

### Transmission Raman measurements

The measurements of TRS signals were performed at 808 nm excitation wavelength, selected as optimum for these tissues elsewhere^14^. The laser power, measured with the power meter after the laser-line filters was maintained constant at 1.3 W during all measurements. The acquisition times of the spectra varied between 2 s with 30 accumulations and 8 s with 10 accumulations. These were chosen to obtain optimum signals whilst avoiding CCD detector saturation.

### Temperature monitoring

Temperature monitoring was performed using a Thermocouple Data Logger (Pico Technology, Cambridgeshire, UK) via 4 channels using probes located in the thermal bath, the quartz vial outside the tissue and inside the tissue.

### Data analysis

All the recorded spectra were imported into Matlab for data pre-processing, which consisted of cosmic background removal, noise filtering using singular value decomposition, background subtraction (first order polynomial) and detector intensity offset removal. Additional peak fitting of the Raman bands intensities were performed using Gaussian peak fitting in Origin (OriginLab, Northampton, USA) following a first order polynomial baseline subtraction.

## Results and discussions

### Tissue attenuation profile

**Figure 2:**
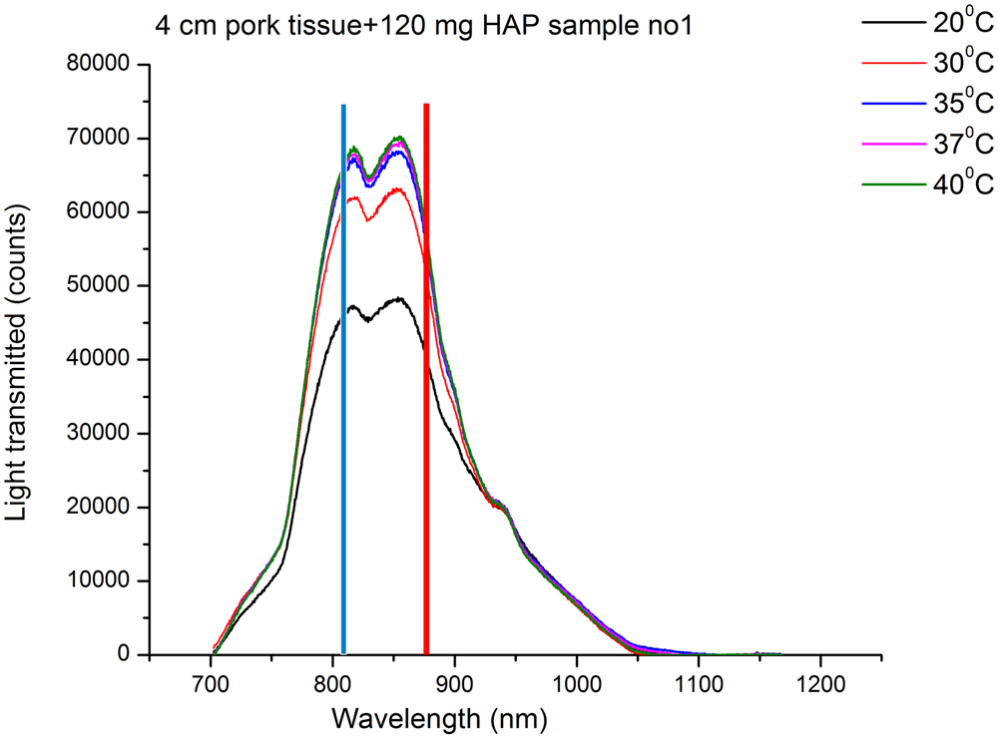
Broadband light transmission spectra of 40 mm tissue (with 120 mg HAP inside the tissue). The marked vertical bands are laser excitation wavelength (blue) and corresponding Raman band of HAP at ~960 cm^−1^ (red). Note the change from 20–30 ^o^C is more significant that the change observed from 30–40 ^o^C.

Figure 2 presents the evolution of the transmission profiles of porcine tissue, with 120 mg HAP powder samples located within the tissue, in the temperature range of 20–40 °C. The blue and red lines mark laser excitation wavelength and corresponding Raman peak of HAP (~960 cm^−1^), respectively. The data provides a direct indication of temperature spectral effects at both the laser and Raman wavelengths. It is evident that more laser photons can travel deeper into tissue and the Raman (signal) photons more easily escape  as a result of the overall increase of transparency at elevated temperatures. Thus by increasing tissue temperature from room to human body temperature, one would expect to observe a higher level of TRS signal leading to consequential higher detection sensitivity.

The observed spectra are dominated by the absorption peaks of fat and water^17^. As the temperature of the tissue is elevated from 20 to 30 °C, we observe a ~30% increase in transmission at the relevant wavelengths. A less significant increase is observed by further elevation to 37 °C/40 °C. This behaviour is likely to be related to the melting of numerous lipids at various temperature points and associate change of their scattering properties^31,32^. Porcine adipose tissue exhibits a heat induced change around 26 °C and 35 °C^32^. Subcutaneous fats have phase changes between 26 °C and 47 °C^32^ with the most significant around 31 °C.

All the transmission measurements of tissue with inclusions of either 120 mg or 80 mg HAP indicate a broadly consistent evolution of the peak heights with temperature for each HAP amount with insignificant influence of HAP powder presence (supporting information Fig. S1b). This is consistent with the fact that the tiny amounts of HAP would not be expected to affect the measured transmission profiles to any significant degree. This is also confirmed through similar data obtained from tissue alone (Fig. S2b).

### Temperature dependence of TRS of porcine tissue

**Figure 3:**
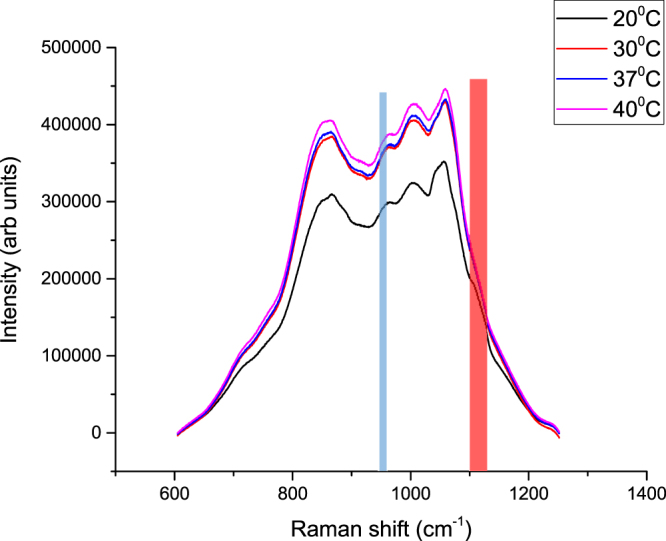
TRS spectra of 40 mm thick slab of porcine tissue at four representative temperatures. The blue band indicates the position of the 960 cm^−1^ Raman peak of HAP, when excitation wavelength is 808 nm. The red highlighted strip indicates 1120 cm^−1^ position, which is the location of a Raman band shown in Fig. 4 to vary with temperature.

As anticipated the TRS measurements carried out on 40 mm-thick porcine tissue at different temperatures reveal a significant enhancement of TRS signals at elevated temperatures from 20 to 40 °C, a type of behaviour not reported previously (Fig. 3). The Raman spectral evolution with temperature is consistent with literature data on tissue absorption profile changes with temperature, as well as our above findings, evidencing increased light transmission through thick biological tissue at both the laser excitation and Raman wavelengths with elevated temperatures above the room temperature.

**Figure 4:**
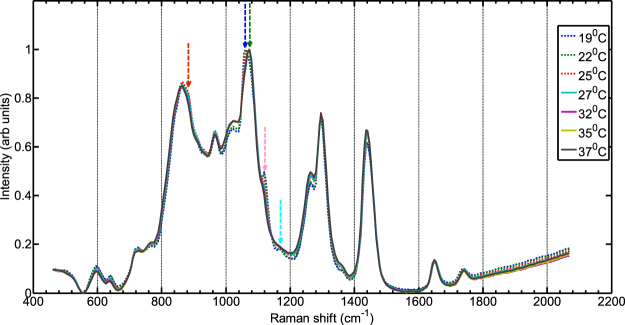
Normalised transmission Raman spectra of the porcine adipose tissue measured with the low dispersion grating and 50 µm slit.

Interestingly, we have also observed the presence of a small Raman band (marked with red highlighted strip) around 1120 cm^−1^ at 20° C, which disappears at higher temperature. The band has been observed consistently in other porcine samples measured under similar conditions. Because of the lower spectral resolution and narrower spectral coverage, we have also performed additional temperature dependence experiments using a low dispersion grating and 50 microns slit on adipose tissue. Figure 4 confirms the presence of the same band at low temperatures and its disappearance at higher temperatures. The data also evidences a shift of the band from ~1060 cm^−1^ (19 °C) to ~1070 cm^−1^ (35 °C). Overall, we observe tissue Raman bands which are typically present at ambient temperature: 883 cm^−1^ -red arrow, 1119 cm^−1^ -pink arrow corresponding to C-C stretching of oleic acid and triglycerides^33^, 1062 cm^−1^ -blue arrow, and 1175 cm^−1^ -arrow of palmitic acids saturated fat^33^. Porcine adipose tissue contains a high percentage of polyunsaturated fat^34^, since several peaks associated with oleic acids are present. As the temperature increases a band shift from 1062 cm^−1^ -blue arrow (C–C aliphatic out-of-phase stretch of triglycerides^35^) towards 1073 cm^−1^ -green arrow while the rest of the bands mentioned above, completely vanish over 30 °C indicating a change in structure of the lipids from gel to liquid configuration. A zoom into the spectral region of 800 cm^−1^–1100 cm^−1^ is presented in Fig. S3; the entire spectral region exhibits an increase in intensity with increasing temperature. We were unable to ascertain whether Raman signal from lipids was higher or lower in absolute terms.

**Figure 5:**
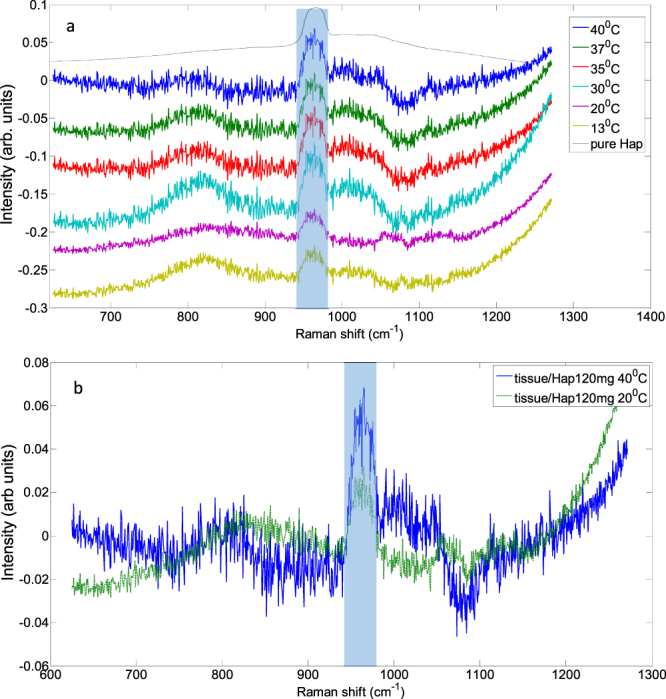
(**a**) Spectral difference of Raman spectra of porcine tissue Raman spectra of porcine tissue with HAP (120 mg) and without HAP at several temperature values (**b**) overlapped plots of spectral difference of Raman spectra of porcine tissue Raman spectra of porcine tissue/120 mg HAP 20 °C and 40 °C.

We tested our hypothesis of improved sensitivity of TRS detection of calcifications in samples at elevated temperatures within a 40 mm thick porcine sample containing 120 mg HAP at its centre. To visualise the weak HAP band we subtracted the TRS signal obtained with tissue alone from that measured with HAP 120 mg within it. The HAP was contained within a quartz vial. The cell was placed inside the tissue in its centre and kept at the same position throughout the measurements. The HAP powder was wrapped in ‘cling film’ and inserted in the cell without disturbing surrounding tissue; thus ensuring a high reproducibility between measurements. Difference spectra plotted in a spectral region around the 960 cm^−1^ HAP band are presented in Fig. 5a. The observed differences evidence the expected evolution of the HAP peak in tissue with temperature. As expected, the biggest jump in HAP Raman intensity is observed between 20 °C and 30 °C. Further elevation from 30 to 40 °C appears to have much smaller influence on the HAP band intensity. The overlap of two difference spectra obtained at 40 °C and 20 °C in Fig. 5b shows more clearly the enhancement of the HAP band intensity with increased temperature. The peak analysis using Gaussian peak fitting yielded a two-fold increase in the peak area between the two temperature points.

The measurements were repeated on other tissue samples (example in Fig. S2). The spectra appear to be noisier, as the changes in peak height/area were less pronounced, an indication of a more scattering sample and explained by tissue variability between two different batches of porcine meat. Overlap of spectral differences at the same temperatures 20 °C and 40 °C are consistent with the results derived from the first sample although the band profiles are less distinguishable due to lower SNR. The estimated gain in TRS signal intensity in this sample was around 30%.

In conclusion we described the effect of temperature on TRS signal strength within both the tissue matrix and any inclusions buried within it. The temperatures explored ranged from room to normal body temperatures (35–37 °C). The smallest recorded value of the TRS signal enhancement in 40 mm thick porcine tissue samples was 30%. The changes are ascribed to temperature specific phase transitions of glycolipids and a consequential reduction of the overall tissue scattering coefficient.

## Electronic supplementary material

Supplementary information

## References

[1] Bruner A, Sutker W, Maxwell G (2009). Minimizing patient exposure to ionizing radiation from computed tomography scans. Proc (Bayl Univ Med Cent).

[2] Stevens O, Iping Petterson IE, Day JC, Stone N (2016). Developing fibre optic Raman probes for applications in clinical spectroscopy. Chem Soc Rev.

[3] Lin K, Zheng W, Lim CM, Huang Z (2016). Real-time *in vivo* diagnosis of laryngeal carcinoma with rapid fiber-optic Raman spectroscopy. Biomed Opt Express.

[4] Lin K (2016). Rapid Fiber-optic Raman Spectroscopy for Real-Time *In Vivo* Detection of Gastric Intestinal Metaplasia during Clinical Gastroscopy. Cancer Prev Res (Phila).

[5] Petterson IEI, Day JCC, Fullwood LM, Gardner B, Stone N (2015). Characterisation of a fibre optic Raman probe within a hypodermic needle. Anal Bioanal Chem.

[6] Wang J (2016). Fiber-optic Raman spectroscopy for *in vivo* diagnosis of gastric dysplasia. Faraday Discuss.

[7] Bergholt MS (2016). Simultaneous fingerprint and high-wavenumber fiber-optic Raman spectroscopy enhances real-time *in vivo* diagnosis of adenomatous polyps during colonoscopy. J Biophotonics.

[8] Jacobs L (2008). Positive margins: the challenge continues for breast surgeons. Ann Surg Oncol.

[9] Keller, M. D. *et al*. Development of a spatially offset Raman spectroscopy probe for breast tumor surgical margin evaluation. *J Biomed Opt* **16** Artn 077006 10.1117/1.3600708 (2011). 10.1117/1.3600708 PMC3144975 21806286

[10] Sickles EA (1984). Mammographic features of “early” breast cancer. AJR Am J Roentgenol.

[11] Frappart L (1984). Structure and composition of microcalcifications in benign and malignant lesions of the breast: study by light microscopy, transmission and scanning electron microscopy, microprobe analysis, and X-ray diffraction. Hum Pathol.

[12] Busing CM, Keppler U, Menges V (1981). Differences in Microcalcification in Breast-Tumors. Virchows Archiv a-Pathological Anatomy and Histopathology.

[13] NHS. Breast Screening Programme, England − 2013-14 [NS]. (Health and Social Care Information Centre, NHS, 2015).

[14] Ghita, A., Matousek, P. & Stone, N. High sensitivity non-invasive detection of calcifications deep inside biological tissue using Transmission Raman Spectroscopy. Journal of Biophotonics, n/a-n/a, 10.1002/jbio.201600260 (2017). 10.1002/jbio.201600260 28635141

[15] Matousek P, Stone N (2013). Recent advances in the development of Raman spectroscopy for deep non-invasive medical diagnosis. J Biophotonics.

[16] Kendall C (2009). Vibrational spectroscopy: a clinical tool for cancer diagnostics. Analyst.

[17] Jacques SL (2013). Optical properties of biological tissues: a review. Physics in Medicine and Biology.

[18] Souza GA (2015). Reference breast temperature: proposal of an equation. Einstein (Sao Paulo).

[19] Kim S, Jeong S (2014). Effects of temperature-dependent optical properties on the fluence rate and temperature of biological tissue during low-level laser therapy. Lasers in Medical Science.

[20] Nau, W. H., Roselli, R. J. & Milam, D. F. Measurement of thermal effects on the optical properties of prostate tissue at wavelengths of 1,064 and 633 nm. *Lasers in Surgery and Medicine* **24**, 38–47, doi:10.1002/(Sici)1096-910124:1 38::Aid-Lsm7 3.0.Co;2-G (1999). 10.1002/(sici)1096-9101(1999)24:1<38::aid-lsm7>3.0.co;2-g 10037350

[21] Pickering JW, Posthumus P, van Gemert MJ (1994). Continuous measurement of the heat-induced changes in the optical properties (at 1,064 nm) of rat liver. Lasers Surg Med.

[22] Chambettaz F, Weible FM, Salathe RP (1993). Temperature dependence of reflectance and transmittance of the artery exposed to air during laser irradiation. IEEE Trans Biomed Eng.

[23] Derbyshire GJ, Bogen DK, Unger M (1990). Thermally induced optical property changes in myocardium at 1.06 microns. Lasers Surg Med.

[24] Jaywant S (1993). Temperature-Dependent Changes in the Optical-Absorption and Scattering Spectra of Tissues - Correlation with Ultrastructure. Proceedings of Laser-Tissue Interaction Iv.

[25] Laufer J, Simpson R, Kohl M, Essenpreis M, Cope M (1998). Effect of temperature on the optical properties of *ex vivo* human dermis and subdermis. Phys Med Biol.

[26] Cletus B, Kunnemeyer R, Martinsen P, McGlone VA (2010). Temperature-dependent optical properties of Intralipid measured with frequency-domain photon-migration spectroscopy. J Biomed Opt.

[27] Lai, P. X., Xu, X. & Wang, L. H. V. Dependence of optical scattering from Intralipid in gelatin-gel based tissue-mimicking phantoms on mixing temperature and time. *J Biomed Op*t **19**, 10.1117/1.Jbo.19.3.035002 (2014). 10.1117/1.JBO.19.3.035002 PMC3945467 24604534

[28] Cletus, B., Kunnemeyer, R., Martinsen, P. & McGlone, V. A. Temperature-dependent optical properties of Intralipid (R) measured with frequency-domain photon-migration spectroscopy. *J Biomed Opt* **15**, 10.1117/1.3290820 (2010). 10.1117/1.3290820 20210477

[29] Harris JR, Lippman ME, Veronesi U, Willett W (1992). Breast cancer (3). N Engl J Med.

[30] Ghita A, Matousek P, Stone N (2016). Exploring the effect of laser excitation wavelength on signal recovery with deep tissue transmission Raman spectroscopy. Analyst.

[31] van Veen, R. L. P., Sterenborg, H. J. C. M., Pifferi, A., Torricelli, A. & Cubeddu, R. In *Biomedical Topical Meeting*. SF4 (Optical Society of America).

[32] Sasaki K, Mitsumoto M, Nishioka T, Irie M (2006). Differential scanning calorimetry of porcine adipose tissues. Meat Sci.

[33] Czamara K (2015). Raman spectroscopy of lipids: a review. J Raman Spectrosc.

[34] Wood JD (2008). Fat deposition, fatty acid composition and meat quality: A review. Meat Science.

[35] Lyndgaard LB, Sorensen KM, van den Berg F, Engelsen SB (2012). Depth profiling of porcine adipose tissue by Raman spectroscopy. J Raman Spectrosc.

