# Supplementary material for: Sensitivity of Transmission Raman Spectroscopy Signals to Temperature of Biological Tissues
Source: Sci Rep. 2018 May 30;8:8379. doi: 10.1038/s41598-018-25465-x (PMC5976642; doi:10.1038/s41598-018-25465-x)
Supplement: Supplementary file 1 — Supplementary information [file 41598_2018_25465_MOESM1_ESM.doc]

Supporting information: Sensitivity of Transmission Raman Spectroscopy Signals to Temperature of Biological Tissues

Adrian Ghita, Pavel Matousek and Nick Stone


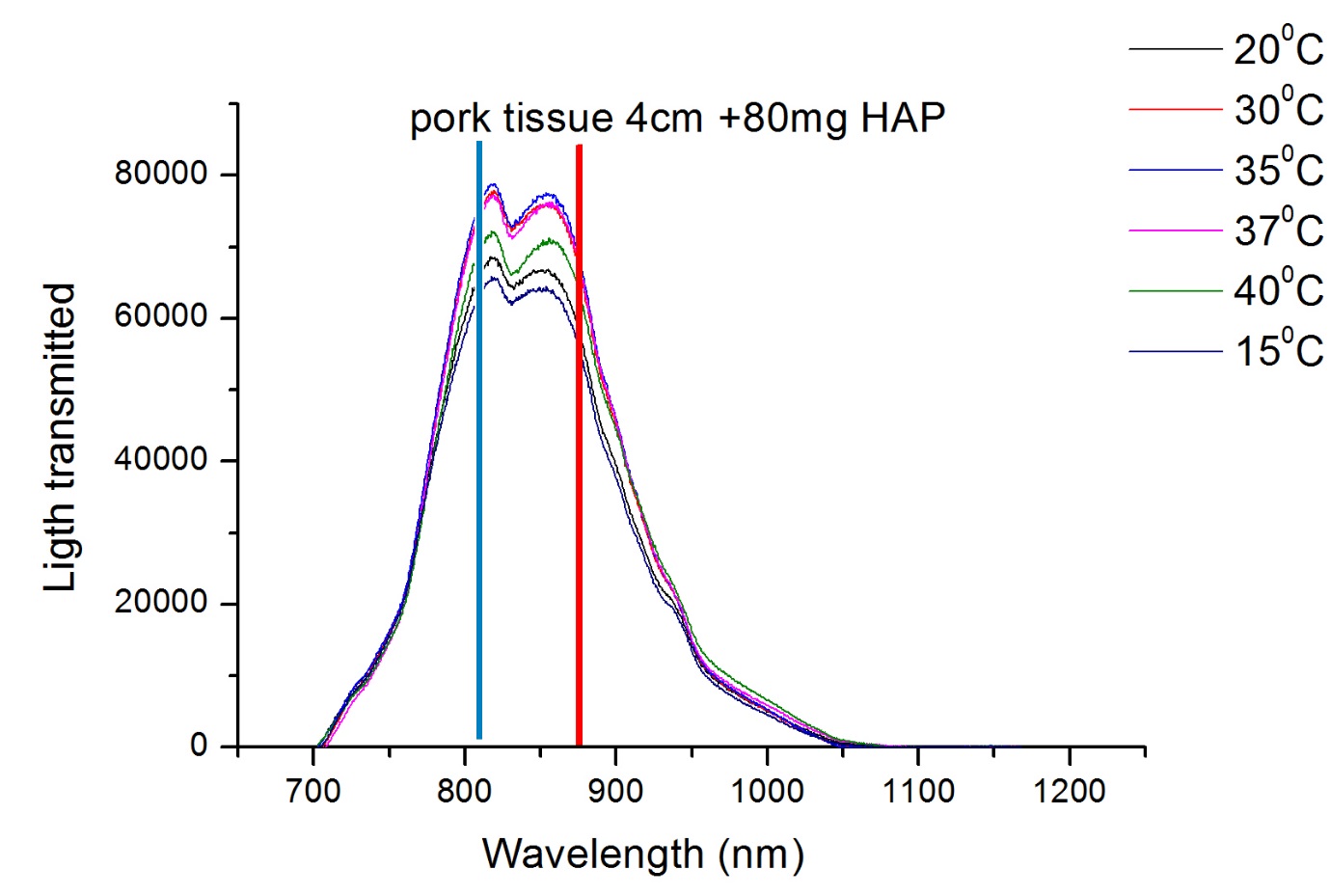


a


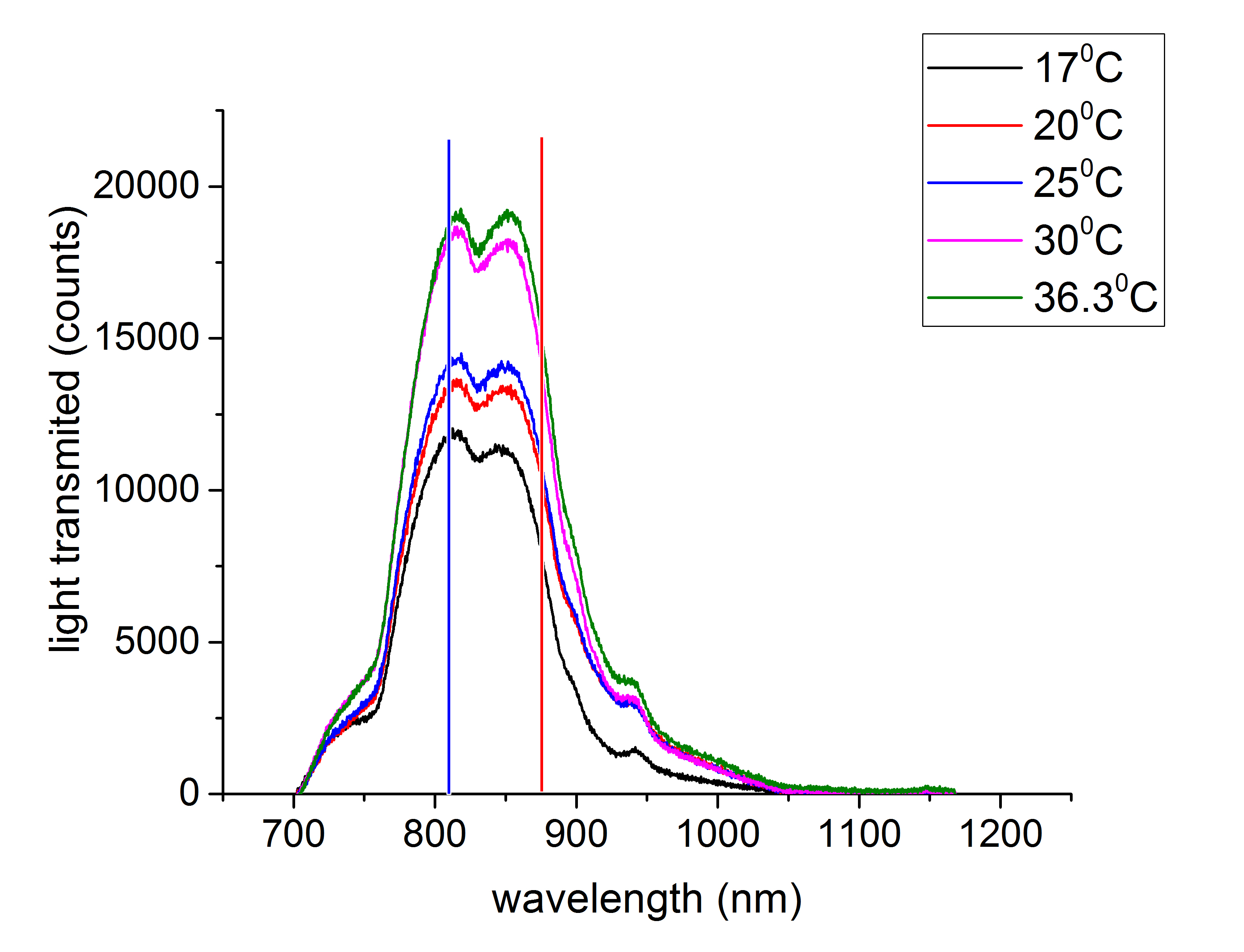


b

**Figure S1:** broadband light transmission spectra of a) 40 mm tissue with 80 mg HAP inside tissue b) 35 mm tissue without HAP.

a

b

**Figure S2**: a) spectral difference of Raman spectra of porcine tissue Raman spectra of porcine tissue with and without HAP (120mg) at several temperature values b) overlapped plots of spectral difference of Raman spectra of porcine tissue Raman spectra of porcine tissue/120mg HAP 20^0^C and 40^0^C.

**Figure S3**: zoom into region 800 cm^-1^ 1100 cm^-1^ from figure 4 (spectra are normalised and baseline subtracted)
